# Serum procalcitonin and procalcitonin clearance as a prognostic biomarker of sepsis in a pediatric critical care setting: A tertiary care experience 2016–2021

**DOI:** 10.1371/journal.pone.0324980

**Published:** 2025-05-22

**Authors:** Rattapon Uppala, Phanthila Sitthikarnkha, Apichaya Kriengwatanasiri, Leelawadee Techasatian, Suchaorn Saengnipanthkul, Sirapoom Niamsanit, Pope Kosalaraksa

**Affiliations:** 1 Department of Pediatrics, Faculty of Medicine, Khon Kaen University, Khon Kaen, Thailand; 2 Research Program in Pediatrics, Khon Kaen University, Khon Kaen, Thailand; Children's National Hospital, George Washington University, UNITED STATES OF AMERICA

## Abstract

**Background:**

Sepsis remains one of the leading causes of morbidity and mortality in critically ill children worldwide. Identifying reliable prognostic markers is essential for improving risk stratification and guiding targeted therapies. While some studies in adults suggest that procalcitonin (PCT) clearance may better predict sepsis outcomes, there is limited information regarding pediatric sepsis. This study aimed to evaluate whether PCT clearance is associated with mortality among children with severe sepsis and septic shock.

**Methods:**

We retrospectively reviewed medical records of children aged 1 month to 18 years admitted to the PICU who were diagnosed with severe sepsis and septic shock at Srinagarind Hospital, Thailand, between January 2016 and October 2021. Serum PCT was measured at 0, 24, and 48 hours after the initial diagnosis. PCT clearance was calculated using the relative change from baseline. The primary outcome was in-hospital mortality.

**Results:**

A total of 242 children were included, with a median age of 8 years (interquartile range [IQR]: 3–14). Most participants (62.8%) had no underlying conditions. The overall mortality rate was 26.5%. An initial PCT level > 2 ng/mL was not significantly associated with mortality (adjusted odds ratio [aOR] = 1.17; 95% confidence interval [CI]: 0.44–3.16, *p* = 0.8). However, decreased PCT clearance at 24 hours was strongly associated with mortality (aOR = 2.79; 95% CI: 1.11–7.01, *p* = 0.029). The area under the receiver operating characteristic curve for 24-hour PCT clearance to predict mortality was 0.71 (95% CI: 0.63–0.80).

**Conclusions:**

Lower PCT clearance in the first 24 hours was significantly associated with higher mortality in pediatric patients with sepsis. Serial PCT measurements and PCT clearance monitoring may offer valuable prognostic information and could be considered as part of routine clinical evaluations in pediatric sepsis management.

## Introduction

Sepsis, defined as life-threatening organ dysfunction arising from a dysregulated host response to infection, continues to be a significant cause of pediatric intensive care unit (PICU) admissions worldwide [1–3]. Although the evolution of pediatric sepsis bundles and best-practice guidelines, partly informed by the Surviving Sepsis Campaign, has led to improvements in recognition and management, mortality and long-term morbidity remain alarmingly high, particularly in resource-limited settings [4,5].

Early identification of high-risk pediatric patients is critical, allowing for prompt, targeted interventions to improve outcomes. Although blood culture is considered the gold standard for the diagnosis of sepsis, its efficacy is constrained by suboptimal sensitivity and prolonged turnaround times [6,7]. Various biomarkers, including C-reactive protein (CRP), lactate, and procalcitonin (PCT) have been explored for their prognostic utility [8]. Lactate is frequently used as a measure of tissue hypoperfusion; however, its levels can be confounded by factors such as liver dysfunction, anemia, seizures, or certain medications, potentially reducing its specificity for sepsis-related risk stratification [9,10]. These limitations have shifted attention toward procalcitonin, a peptide precursor of calcitonin released in response to bacterial infections and often correlates with infection severity.

Recent adult studies suggest that changes in PCT levels over time, termed PCT clearance, can offer better prognostic insights than a single PCT measurement alone. Persistently elevated or slowly declining PCT might indicate inadequate source control, ongoing infection, or a robust inflammatory response, all of which could correlate with worse outcomes [11–14]. Despite growing evidence in adults, there is a paucity of data regarding PCT clearance in pediatric populations with sepsis and septic shock.

To address this gap, we conducted a retrospective cohort study in children admitted with severe sepsis and septic shock to assess the relationship between PCT clearance and mortality. We hypothesized that slower PCT clearance within the first 24–48 hours would be associated with a higher risk of mortality.

## Materials and methods

### Study design and population

We performed a retrospective cohort study including children aged 1 month to 18 years who were diagnosed with severe sepsis or septic shock and admitted to the PICU at Srinagarind Hospital, Khon Kaen University, from January 2016 through October 2021. Patients with cyanotic congenital heart disease, end-stage malignancy, terminal illnesses, or significant congenital anomalies were excluded. The study adhered to the Strengthening the Reporting of Observational Studies in Epidemiology (STROBE) guidelines. Upon receiving ethical approval from the institutional review board, we initiated a review of the medical records belonging to pediatric patients who met the inclusion criteria. This data collection process encompassed from December 15, 2022, to November 30, 2023.

At our institution, children admitted with severe sepsis or septic shock typically undergo PCT testing at presentation and at 24, 48, and 72 hours thereafter, in line with local protocols for infection and sepsis monitoring.

### Definitions

Severe sepsis and septic shock were defined according to the International Pediatric Sepsis Consensus Conference criteria [15]. Severe sepsis: Sepsis plus at least one of the following: cardiovascular dysfunction, acute respiratory distress syndrome (ARDS), or ≥ 2 other organ system dysfunctions. Septic shock: Sepsis with cardiovascular dysfunction requiring vasoactive medications or fluid resuscitation support.

### Data collection

We gathered baseline patient information, which included age, sex, weight, height, and comorbidities. We categorized the children into five distinct groups for the analysis process as follows: 1) infants (<1 year), 2) toddlers (1 to <3 years), 3) preschool-aged children (3 to <5 years), 4) school-aged children (5–12 years), and 5) adolescents (>12–18 years). Laboratory results were obtained from electronic medical records and comprised a complete blood count, serum urea, creatinine, lactate, and PCT. The PCT levels were documented according to the institution’s protocol for children diagnosed with sepsis and septic shock. Measurements of PCT levels were performed using an electrochemiluminescence immunoassay (Cobas E 801 analyzer, Roche, Japan) and reported in nanograms per milliliter (ng/mL). The PCT clearance was determined by calculating the difference between the initial PCT and the PCT levels measured at 24, 48, and 72 hours. This difference was then divided by the initial PCT value and expressed as a percentage.

The clinical outcomes of interest included two key factors: the rate of in-hospital mortality and the length of hospital stay. These metrics provide valuable insights into patient health status and the effectiveness of care received during hospitalization.

### Statistical analysis

All analyses were conducted using Stata software version 10 (StataCorp LP, College Station, TX, USA). Categorical data were expressed as frequencies (percentages), while continuous data were summarized as means ± standard deviation (SD) or medians (interquartile range [IQR]), depending on distribution. Univariate logistic regression was used to identify factors associated with in-hospital mortality; results are presented as crude odds ratios (OR) with 95% confidence intervals (CI). Statistically significant variables (p < 0.2) in the univariate analysis were then included in a multivariable logistic regression using a backward stepwise approach. Adjusted odds ratios (aOR) and 95% CIs were calculated for factors retained in the final model. The predictive performance of PCT clearance was assessed by generating a receiver operating characteristic (ROC) curve and calculating the area under the ROC (AUROC). A *p*-value < 0.05 was considered statistically significant.

### Ethical approval

This study was approved by the Khon Kaen University Ethics Committee (KKUEC), approval number HE641625. The Institutional Review Board waived the requirement for written informed consent in accordance with Khon Kaen University’s Announcement No. 2179/2563, as it involved retrospective analysis of existing data without identifying individual participants.

## Results

### Studied population and characteristics

**Fig 1:**
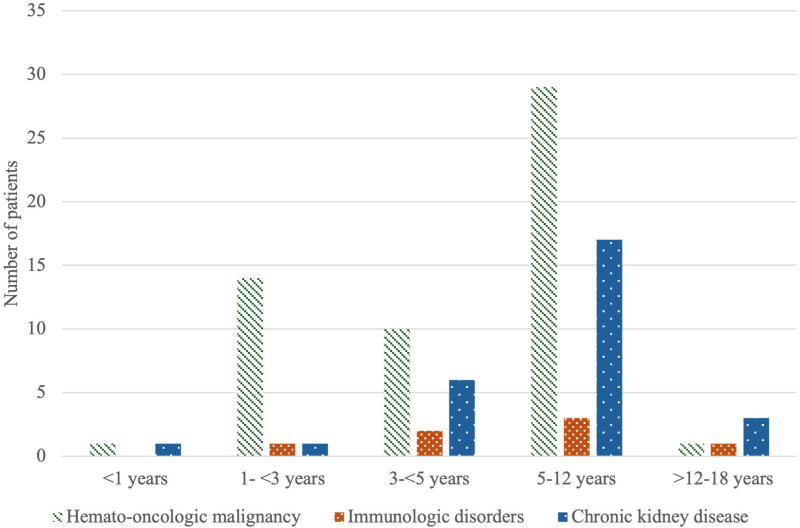
The distribution of preexisting co-morbidities across each age group among children with severe sepsis or septic shock admitted to the pediatric intensive care unit.

A total of 242 children with severe sepsis or septic shock were included. Table 1 summarizes their baseline characteristics. The median age was 8 years (IQR: 3–14), and 55.8% were female. Most patients (62.8%) had no known comorbidities. (Table 1) Among those with underlying illnesses, hemato-oncologic malignancies (22.7%) were most common, followed by immunologic disorders (11.6%) and chronic kidney disease (2.9%). The distribution of preexisting co-morbidities across each age group was illustrated in Fig 1. The overall in-hospital mortality was 26.5%.

**Table 1:** Demographic characteristics of children with severe sepsis or septic shock admitted to the pediatric intensive care unit.

Characteristics	Total (N = 242)	Death (N = 64)	Survivors (N = 178)
**Gender, n (%)**			
Male	107 (44.2)	27 (42.2)	80 (44.9)
Female	135 (55.8)	37 (57.8)	98 (55.1)
**Age (years), median (IQR)**	5.3 (1.2-12.4)	2.5 (0.5-10.8)	7 (1.8-12.8)
**Preexisting co-morbidities, n (%)**			
No underlying disease	152 (62.8)	42 (65.6)	110 (61.8)
Hemato-oncologic malignancy	55 (22.7)	16 (25)	39 (21.9)
Immunologic disorders	28 (11.6)	1 (1.56)	6 (3.4)
Chronic kidney disease	7 (2.9)	5 (7.8)	23 (7.8)
**Length of stay, median (IQR)**	20 (9-42)	14.5 (5-35)	23 (10-47)

### Procalcitonin levels, PCT clearance, and mortality

Serum PCT levels were measured at admission (0 hours) and 24 hours in all 242 patients; 146 had additional measurements at 48 hours. The median (IQR) initial PCT and lactate levels were 0.625 ng/mL (0.043–100) and 15.8 mg/dL (5.8–154.6), respectively.

### Factors associated with mortality of severe sepsis or septic shock

**Fig 2:**
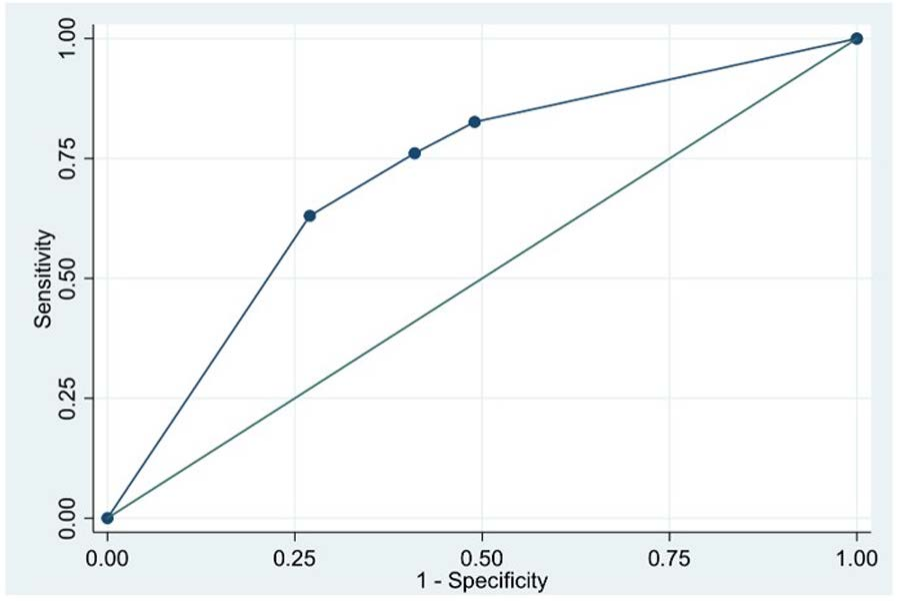
Receiver Operating Characteristic (ROC) Curve for 24-hour PCT clearance in predicting mortality among children with severe sepsis or septic shock admitted to the pediatric intensive care unit.

An initial PCT concentration > 2 ng/mL was not significantly associated with mortality (OR = 1.17; 95% CI: 0.44–3.16, p = 0.8). By contrast, patients with an initial lactate > 4 mg/dL were at significantly higher risk of death (aOR = 2.80; 95% CI: 1.23–6.41, p = 0.015). Notably, reduced PCT clearance at 24 hours was strongly linked to higher mortality (aOR = 2.79; 95% CI: 1.11–7.01, p = 0.029) (Table 2). The AUROC for 24-hour PCT clearance to predict mortality was 0.71 (95% CI: 0.63–0.80), indicating acceptable discrimination (Fig 2).

**Table 2:** Factors associated with mortality of severe sepsis or septic shock in children admitted to the pediatric intensive care unit.

Variables	Outcome		Crude odds ratio	Adjusted odds ratio	95%CI	p-value
	Death (N = 64)	Survivors (N = 178)				
**Age**						
<1 years	22 (34.38)	31 (17.42)	Reference	Reference		
1- < 3 years	12 (18.75)	23 (12.92)	0.74	0.73	0.28-1.92	0.520
3- < 5 years	7 (10.94)	20 (11.24)	0.49	0.81	0.27-2.47	0.717
5-12 years	12 (18.75)	62 (34.83)	0.27	0.35	0.15-0.84	0.019
>12–18 years	11 (17.19)	42 (23.60)	0.37	0.40	0.16-0.89	0.048
**Initial lactate**						
< 2 mmol/L	23 (35.94)	55 (30.90)	Reference	Reference		
2-4 mmol/L	9 (14.06)	17 (9.55)	1.27	1.21	0.46-3.17	0.700
> 4 mmol/L	22 (34.38)	17 (9.55)	3.09	2.80	1.23-6.41	0.015
**Procalcitonin clearance at 24 hours**						
Increase	11 (23.91)	59 (59.00)	Reference	Reference		
Decrease	35 (76.09)	41 (41.00)	4.58	2.79	1.11-7.01	0.029

## Discussion

In this retrospective study of pediatric severe sepsis and septic shock, we found that initial serum PCT levels alone did not correlate significantly with mortality. In contrast, early blood lactate >4 mg/dL was associated with a higher risk of death, consistent with the well-documented link between hyperlactatemia and poor outcomes in sepsis [9,10]. Notably, a reduction in PCT clearance by 24 hours was independently associated with higher mortality, reinforcing the clinical relevance of dynamic PCT trends in pediatric sepsis.

These findings align with reports from adult populations, where PCT clearance has been identified as a more reliable prognostic marker than a single PCT measurement [11,14]. Slower or absent declines in PCT may signify ongoing inflammation or suboptimal source control, factors that can lead to worse outcomes [16]. While PCT has become a useful tool in some adult sepsis protocols, data in pediatric cohorts remain limited. Our study contributes evidence that PCT clearance can similarly stratify risk among critically ill children with sepsis.

Clinically, PCT clearance could serve as an adjunctive measure to track response to therapy [17]. Under conditions of sepsis, microbes and their antigens activate a variety of anti-inflammatory mediators, which subsequently initiate the host immune response [18]. Therefore, PCT serves as a valuable tool for monitoring antimicrobial treatment protocols. Persistently elevated or increasing levels of PCT may encourage clinicians to reevaluate the antimicrobial regimen, search for undiscovered sources of infection, or escalate supportive care. Conversely, a rapid decline in PCT could support de-escalation strategies, especially in settings where antibiotic stewardship is a priority [19]. However, our data also highlights that PCT alone cannot be used in isolation. Lactate and other clinical parameters remain invaluable, and future research could explore combined biomarker approaches.

Several limitations warrant consideration. First, this single-center study may not capture the full heterogeneity of pediatric sepsis, limiting the generalizability of our findings. Second, some children received antibiotics before transferring to our tertiary care center, potentially influencing baseline PCT levels and blood culture results. Third, we did not evaluate daily severity of illness scores (e.g., PRISM-III) for correlation with PCT dynamics. Finally, incomplete PCT measurements at 48 or 72 hours in some patients reduced our sample size for later time points. Further prospective, multi-center studies are needed to confirm the prognostic utility of PCT clearance and to define optimal threshold values. Investigations integrating PCT clearance with established severity-of-illness scores or with other biomarkers such as CRP and lactate may offer more comprehensive risk stratification models. In this study, we focused on in-hospital mortality rather than LOS; therefore, we did not analyze the correlation between initial PCT levels and LOS. We acknowledge this as a limitation, and future research should investigate the potential associations between PCT levels, LOS, and treatment effectiveness to further clarify the clinical utility of PCT. Patients with immune disorders included individuals across various age groups. Due to a limited sample size, we did not analyze PCT clearance specifically by age for this subgroup. Future research should further explore subgroup differences in PCT clearance to enhance our understanding of clinical outcomes. Additionally, randomized controlled trials examining how PCT-guided therapeutic modifications affect clinical outcomes in pediatric sepsis could yield important insights.

## Conclusion

Our study provides evidence that a decline in PCT clearance within the first 24 hours of severe sepsis or septic shock is significantly associated with mortality in children. Although an initial PCT measurement alone did not predict outcomes, serial PCT assessments and trends offer meaningful prognostic information. Monitoring PCT kinetics may help guide clinical decision-making and highlight patients at heightened risk but should be used in conjunction with other clinical and biochemical markers for a comprehensive evaluation.

## References

[1] Kissoon N, Carcillo JA, Espinosa V, Argent A, Devictor D, Madden M, et al. World Federation of Pediatric Intensive Care and Critical Care Societies: Global Sepsis Initiative. Pediatr Crit Care Med. 2011 Sep ;12(5):494–503. 21897156 10.1097/PCC.0b013e318207096c

[2] Brierley J, Carcillo JA, Choong K, Cornell T, Decaen A, Deymann A, et al. Clinical practice parameters for hemodynamic support of pediatric and neonatal septic shock: 2007 update from the American College of Critical Care Medicine. Crit Care Med. 2009;37(2):666–88. doi: 10.1097/CCM.0b013e31819323c6 19325359 PMC4447433

[3] Fleischmann-Struzek C, Goldfarb DM, Schlattmann P, Schlapbach LJ, Reinhart K, Kissoon N. The global burden of paediatric and neonatal sepsis: a systematic review. Lancet Respir Med. 2018;6(3):223–30. doi: 10.1016/S2213-2600(18)30063-8 29508706

[4] Samransamruajkit R, Limprayoon K, Lertbunrian R, Uppala R, Samathakanee C, Jetanachai P, et al. The Utilization of the Surviving Sepsis Campaign Care Bundles in the Treatment of Pediatric Patients with Severe Sepsis or Septic Shock in a Resource-Limited Environment: A Prospective Multicenter Trial. Indian J Crit Care Med. 2018;22(12):846–51. doi: 10.4103/ijccm.IJCCM_367_18 30662223 PMC6311978

[5] Rivers E, Nguyen B, Havstad S, Ressler J, Muzzin A, Knoblich B. Early goal-directed therapy in the treatment of severe sepsis and septic shock. N Engl J Med. 2001;345(19):1368–77. 11794169 10.1056/NEJMoa010307

[6] Liu BM, Carlisle CP, Fisher MA, Shakir SM. The brief case: Capnocytophaga sputigena bacteremia in a 94-year-old male with type 2 diabetes mellitus, pancytopenia, and bronchopneumonia. J Clin Microbiol. 2021;59(7):e0247220. 10.1128/JCM.02472-20 PMC8218739 34142857

[7] Duncan CF, Youngstein T, Kirrane MD, Lonsdale DO. Diagnostic Challenges in Sepsis. Curr Infect Dis Rep. 2021;23(12):22. doi: 10.1007/s11908-021-00765-y 34720754 PMC8544629

[8] Silvestre J, Póvoa P, Coelho L, Almeida E, Moreira P, Fernandes A, et al. Is C-reactive protein a good prognostic marker in septic patients?. Intensive Care Med. 2009;35(5):909–13. doi: 10.1007/s00134-009-1402-y 19169668

[9] Kim YA, Ha E-J, Jhang WK, Park SJ. Early blood lactate area as a prognostic marker in pediatric septic shock. Intensive Care Med. 2013;39(10):1818–23. doi: 10.1007/s00134-013-2959-z 23818093

[10] Suetrong B, Walley KR. Lactic Acidosis in Sepsis: It’s Not All Anaerobic: Implications for Diagnosis and Management. Chest. 2016;149(1):252–61. doi: 10.1378/chest.15-1703 26378980

[11] Mat Nor MB, Md Ralib A. Procalcitonin clearance for early prediction of survival in critically ill patients with severe sepsis. Crit Care Res Pract. 2014;2014:819034. doi: 10.1155/2014/819034 24719759 PMC3955692

[12] Jain S, Sinha S, Sharma SK, Samantaray JC, Aggrawal P, Vikram NK, et al. Procalcitonin as a prognostic marker for sepsis: a prospective observational study. BMC Res Notes. 2014;7:458. doi: 10.1186/1756-0500-7-458 25034373 PMC4105100

[13] Rey C, Los Arcos M, Concha A, Medina A, Prieto S, Martinez P, et al. Procalcitonin and C-reactive protein as markers of systemic inflammatory response syndrome severity in critically ill children. Intensive Care Med. 2007;33(3):477–84. doi: 10.1007/s00134-006-0509-7 17260130

[14] Ruiz-Rodríguez JC, Caballero J, Ruiz-Sanmartin A, Ribas VJ, Pérez M, Bóveda JL, et al. Usefulness of procalcitonin clearance as a prognostic biomarker in septic shock. A prospective pilot study. Med Intensiva. 2012;36(7):475–80. doi: 10.1016/j.medin.2011.11.024 22257436

[15] Goldstein B, Giroir B, Randolph A, International Consensus Conference on Pediatric Sepsis. International pediatric sepsis consensus conference: definitions for sepsis and organ dysfunction in pediatrics. Pediatr Crit Care Med. 2005;6(1):2–8. doi: 10.1097/01.PCC.0000149131.72248.E6 15636651

[16] Branche A, Neeser O, Mueller B, Schuetz P. Procalcitonin to guide antibiotic decision making. Curr Opin Infect Dis. 2019;32(2):130–5. doi: 10.1097/QCO.0000000000000522 30648993

[17] Gilbert DN. Use of plasma procalcitonin levels as an adjunct to clinical microbiology. J Clin Microbiol. 2010;48(7):2325–9. doi: 10.1128/JCM.00655-10 20421436 PMC2897488

[18] Assicot M, Gendrel D, Carsin H, Raymond J, Guilbaud J, Bohuon C. High serum procalcitonin concentrations in patients with sepsis and infection. Lancet. 1993;341(8844):515–8. 8094770 10.1016/0140-6736(93)90277-N PMC7141580

[19] Schuetz P, Beishuizen A, Broyles M, Ferrer R, Gavazzi G, Gluck EH, et al. Procalcitonin (PCT)-guided antibiotic stewardship: an international experts consensus on optimized clinical use. Clin Chem Lab Med. 2019;57(9):1308–18. doi: 10.1515/cclm-2018-1181 30721141

